# Perception of visual noise patterns depends on correlations produced in V1: A simulation study

**DOI:** 10.1167/jov.26.10.10

**Published:** 2026-10-09

**Authors:** Shaozhi Nie, Samantha A. Montoya, Michael-Paul Schallmo, Stephen A. Engel

**Affiliations:** 1 Department of Psychology, University of Minnesota Twin Cities, Minneapolis, MN, USA; 2 Department of Neurology, University of Pennsylvania, Philadelphia, PA, USA; 3 Department of Psychiatry and Behavioral Sciences, University of Minnesota Twin Cities, Minneapolis, MN, USA

**Keywords:** visual noise, visual snow syndrome, primary visual cortex, computational modeling, correlated activity

## Abstract

Visual noise is widely used as a stimulus in vision science, yet the neural representation of noise itself remains poorly understood. We investigated how external visual noise is encoded in the primary visual cortex (V1) using a biologically inspired neural network model (VOneNet). We simulated V1 responses to noise patterns and analyzed both the response of individual model neurons and the correlations among them. As expected, simulated neurons with smaller receptive fields showed stronger responses. Importantly, spatially independent noise stimuli elicited correlations across the population. These correlations were critical for reconstructing the original noise pattern in a reconstruction analysis that served as a probe of perceptual representation: decorrelating modeled neural activity substantially degraded reconstruction quality. Correlations among simulated simple cells, rather than complex cells, played a dominant role in encoding external noise. Our findings suggest that the perception of visual noise depends not only on local neural activity but also on population-level correlations in V1. Implications for visual snow syndrome, a condition in which illusory external noise is perceived, are discussed.

## Introduction

External visual noise, i.e., random variations in intensity in the visual field, arises in diverse contexts, and has attracted significant interest from researchers. One source of attention has been the use of noise as a stimulus: In psychophysics, it can serve as a masker, or a method for measuring internal perceptual noise (e.g., Bacon-Macé et al., 2005; Gold, Bennett, & Sekuler, 1999; Pelli, Farell, & Moore, 2003; Pelli & Farell, 1999). Reverse correlation methods have also used noise as part of a stimulus to infer mechanisms underlying perception of targets of various types in behavioral studies (Ahumada, 2002; Neri & Levi, 2006). External noise is even more widely used as a stimulus in studies of physiology to characterize neural receptive fields (e.g., Ringach & Shapley, 2004).

Perception of noise itself has been studied much less. One example is in camera design and image encoding, where one may want to minimize the amount of noise perceived in a device's output (Xiao, Farrell, & Wandell, 2005). In neurology and ophthalmology, perception of noise is of recent interest because of the neurological condition known as visual snow syndrome, where people perceive something resembling dynamic visual noise continuously covering the visual field (Metzler & Robertson, 2018; Fraser, 2022; Montoya et al., 2024; Brooks et al., 2024). Although visual snow is generated internally, recent behavioral findings suggest that its neural representation may at least partially overlap with representations generated by externally presented visual noise (Montoya, Mulder, Lee, Schallmo, & Engel, 2023; Montoya et al., 2025).

Here we are concerned with the perception of external noise patterns, and in particular how external noise is encoded across the population of neurons in primary visual cortex. Two questions immediately arise, based on first and second order statistics of the population response. The first is: Which neurons respond most strongly to a noise stimulus?

Logically, noise should generate stronger signals in smaller receptive fields or subfields if inputs are pooled linearly, other things being held equal, because random variations will tend to cancel each other more across larger areas. But to our knowledge this observation has not been tested empirically. Furthermore, an implicit assumption in using *white* noise is that because it contains equal energy across the Fourier spectrum, one might expect it to produce equivalent responses across the population of V1 neurons, which are tuned to portions of that spectrum. This intuition is likely to be false for the same reason- smaller receptive fields will show larger variations in response than larger receptive fields. Note that this will be the case if, as is commonly done, the white noise is constructed to have equal energy at different frequencies across the entire image. Then, receptive fields that sample a smaller portion of the image would be expected to have more variability in response, across locations or image samples (again, other things being equal).

The second question is to what extent correlations between cortical neurons are produced by external noise (i.e., second order statistics), and whether these are required for noise perception. One might assume that because noise pixels are generated independently, that they will produce little correlation in neural response. But of course, correlations in receptive field structure (e.g., overlap in which pixels drive them) can produce correlated response from uncorrelated pixels. Whether such correlations are important has been investigated in retinal ganglion cells, where external white noise appears to be encoded primarily with spatial independence: cell-to-cell correlations provide minimal information about noise stimuli (Pillow et al., 2008), though slightly more than when encoding natural images (Meytlis et al., 2012).

Few studies have investigated visual noise representation in cortex. Correlations between responses of V1 neurons may be defining features of naturalistic visual textures, and neurons in V2 appear to be sensitive to them (Freeman, Ziemba, Heeger, Simoncelli, & Movshon, 2013). Noise images in this and related work served as control conditions, and the strength and importance of correlations in them has not been closely examined. Here we measure the extent to which correlations exist for noise images also. Such correlations may allow external visual noise to be processed in the same way as other textures.

Recent advances in artificial neural networks allow us to investigate the representation of visual noise in silico. Biologically inspired neural networks, such as VOneNet (Dapello et al., 2020), have been developed to simulate the early visual system with neurophysiologically realistic receptive field parameters. These models incorporate non-human primate V1-like properties including appropriate distributions of receptive field and subfield sizes, preferred spatial frequencies, and orientations, output nonlinearities, and additional nonlinearities present in complex cells (Rust, Schwartz, Movshon, & Simoncelli, 2005). The networks simulate the responses of thousands of neurons simultaneously while processing complex visual inputs. Furthermore, reconstruction networks allow us to probe what information is preserved in the modeled V1 response, and to estimate how that encoded information may be represented perceptually by downstream visual processing (Parthasarathy et al., 2017; Wu et al., 2024). This reflects the assumption that perception corresponds to the world configuration decoded from a pattern of neural activity.

The present work leverages these computational advances to examine which V1 receptive fields are most important for representing snow-like visual noise, and whether correlations amongst V1 responses are also an important part of the representation. Specifically, we used the relevant layer of VOneNet to simulate the neural responses in V1 (Figure 1; Dapello et al., 2020). We then generated profiles of responses to external noise, and calculated correlations between them. We also built a network to reconstruct visual noise from the simulated V1 neural responses and conducted perturbation analyses to determine the contribution of different neural populations and their correlations. We find that external noise patterns generate the highest level of activities in neurons with smaller receptive fields, as expected. The noise images also produced patterns of correlation across neural populations that preserve crucial information about the visual noise structure.

**Figure 1.**
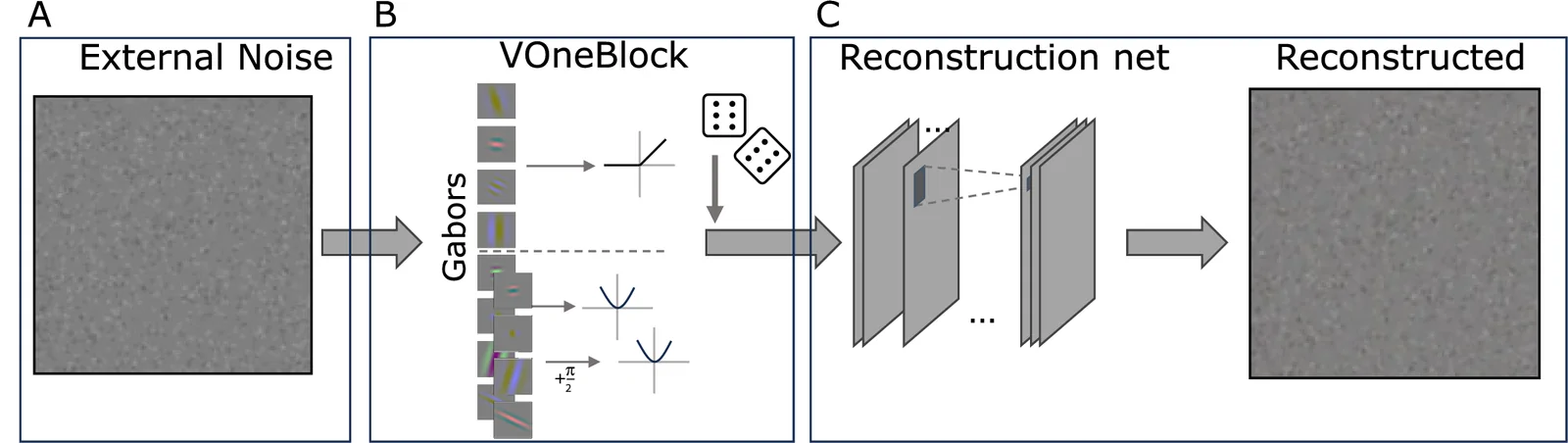
Illustration of the workflow. Our method consisted of three components: (**A**) generation of external noise, (**B**) simulation of V1 responses using VOneBlock; dice represent internal noise added at the end of simulation of V1 neurons, (**C**) image reconstruction.

## Methods

The simulation workflow consisted of three main components (Figure 1), detailed below. First, we generated images containing visual noise, which served as input to a network simulating V1 neurons. The resulting neural responses were then decoded using a reconstruction network, which we treated as a form of perceptual readout. Under this interpretation, reconstructed images provide an estimate of the visual pattern that could be represented perceptually given the encoded V1 activity.

### Generation of the external input noise

We generated visual noise within a simulated visual field of 2.24 × 2.24 degrees, at a resolution of 100 pixels per degree, approximating the density of parafoveal retinal ganglion cells (Wässle, Grünert, Röhrenbeck, & Boycott, 1989). The resulting 224 × 224-pixel images were higher in resolution than what was assumed to be 28 × 28 pixels in the previous modeling paper, Dapello et al. (2020). We increased PPD to allow the simulation of high-resolution visual noise patterns and to align with the input dimensionality. Such change is in the range of V1 neurons studied in the neuroscientific study that provided training data for the network (Rust et al., 2005).

We focused on external noise that resembled “visual snow”, since results based on such noise might have clinical relevance. Visual snow is a newly isolated, surprisingly common condition (>2% prevalence) in which people continuously see tiny low contrast specks flickering throughout the entire visual field (e.g., Costa et al., 2022; Muravikova et al., 2024). The parameters of our noise were drawn from a study in which participants matched the appearance of their visual snow using a simulation (Montoya et al., 2024).

The noise consisted of independently distributed small Gaussian-shaped luminance dots that were either brighter or darker than the background. Each dot was characterized by a two-dimensional Gaussian profile with a standard deviation of 1 pixel (i.e., 0.01 visual degrees). The dots were generated by assigning a percentage of pixels—referred to as the density —a positive or negative luminance increment drawn from a binary distribution. We used a density of 3% to match percepts of visual snow (Montoya et al., 2024). The contrast parameter, which determined the possible luminance increments, was set to 0.63, meaning each noise dot had a luminance value of either −0.63 or 0.63. (For reference, the full range of possible luminance increments was −1 to +1, where −1 represents a black pixel and +1 represents a white pixel.) These dots were then blurred with the 1-pixel Gaussian filter. In short, each pixel had a 3% probability of being the center of a small Gaussian blob, either positive or negative. Despite the low nominal density, the Gaussian blur gave the noise a denser appearance (Figure 1). To enhance generalizability, we used both uniform gray backgrounds and colored natural images (randomly selected from ImageNet; Deng et al., 2009) as the base on which noise was added. Pixel luminance values exceeding the valid range after noise addition were clipped, though this occurred rarely (0.7% color channel values were clipped, and 1.3% pixels had at least one clipped channel).

### Simulation of V1 neurons

We adapted VOneBlock, the first layer of VOneNet (Dapello et al., 2020), an existing CNN that simulated early vision using biologically reasonable parameters, e.g., the distribution of receptive field types. This model makes the common assumption that responses can be captured by linear or nonlinear combinations of filter banks (De Valois, Albrecht, & Thorell, 1982; Vintch, Movshon, & Simoncelli, 2015) placed in a convolutional neural network (CNN, Nguyen, Sooriyaarachchi, Huang, & Baker, 2024; Dapello et al., 2020) followed by a non-linear response function (Figure 1). The simulation had a convolutional layer of 512 “channels”: Half (256) were Gabor-shaped linear filters of various sizes, spatial frequencies, and orientations followed by rectification, corresponding to simple cells. The other half were similar filters in quadrature pairs that were squared and summed, corresponding to complex cells. Each channel was defined by its X-size, Y-size, spatial frequency, orientation, and spatial phase. X- and Y-sizes are standard deviations of the two-dimensional gaussian envelope parallel and orthogonal to the orientation of the Gabor filter.

Across the possible 224 by 224 positions of the neurons, the simulation contained 512 (*channels*) × 224 (*rows*) × 224 (*columns*) = ∼2.6 × 10^7^ neurons. The VOneBlock model also contained terms to simulate internal neuronal noise, by adding draws from an independent gaussian distribution to each neuron, with the variance equal to its activation (Dapello et al., 2020). Such noise is widely used to approximate realistic neural responses and to improve the robustness of the network (Doyle & Evans, 2018).

The original version of the VOneNet (Dapello et al., 2020) contained a down-sampling procedure following the convolution, with a convolutional stride of 4 pixels. To allow more detailed spatial information to be retained, we changed the convolutional stride to 1 pixel, which kept the output of the simulated neural responses the same size as the input image.

### Reconstruction of encoded noise

To investigate the information encoded in the simulated neural responses, we built a network to decode it: A simple CNN, referred to as the reconstruction net, was used to estimate the external input that produced the simulated V1 responses. The reconstruction net consisted of 5 convolutional layers with a rectification unit following each layer. To be consistent with the VOneNet, we trained the reconstruction net with the ImageNet (Deng et al., 2009) images. Since the perceived similarity between images does not merely depend on the pixel-wide similarity but also the similarity in image statistics such as contrast, we used negative multi-scale structural similarity, a human vision-based measure of perceived similarity (Wang, Simoncelli, & Bovik, 2003), as the objective function during training (The weights of the VOneNet were fixed throughout the study). Importantly, the reconstruction net was trained only on unaltered neural responses, and all perturbation manipulations were introduced exclusively at the time of testing to assess their impact on reconstruction performance.

We quantified perceptual similarity using the Portilla–Simoncelli texture model (Portilla & Simoncelli, 2000), which summarizes perceptually relevant texture with joint image statistics. We then measured the Manhattan distance between the statistics of each reconstruction and its original counterpart; larger distances indicated greater changes in reconstructed texture, and a loss of perceptual similarity between the two.

## Results

### Descriptive statistics of the simulated neural responses

Simulated receptive field subfields were larger than the individual visual noise elements, whose size was a Gaussian with a 1-pixel standard deviation (See Methods). Across the population, the mean preferred spatial frequency was one cycle in 5.6 pixels, and the highest was one cycle in 3 pixels.

We first investigated which simulated neurons in our V1 model showed the largest responses to the external noise. We computed the distributions of neural responses of different *channels*, assuming spatial homogeneity, by pooling responses of neurons with identical receptive fields, but at different positions. We also gathered statistics across 40 different noise sample images. Figure 2A plots superimposed histograms of responses for the channels in response to images with versus without added visual noise.

**Figure 2.**
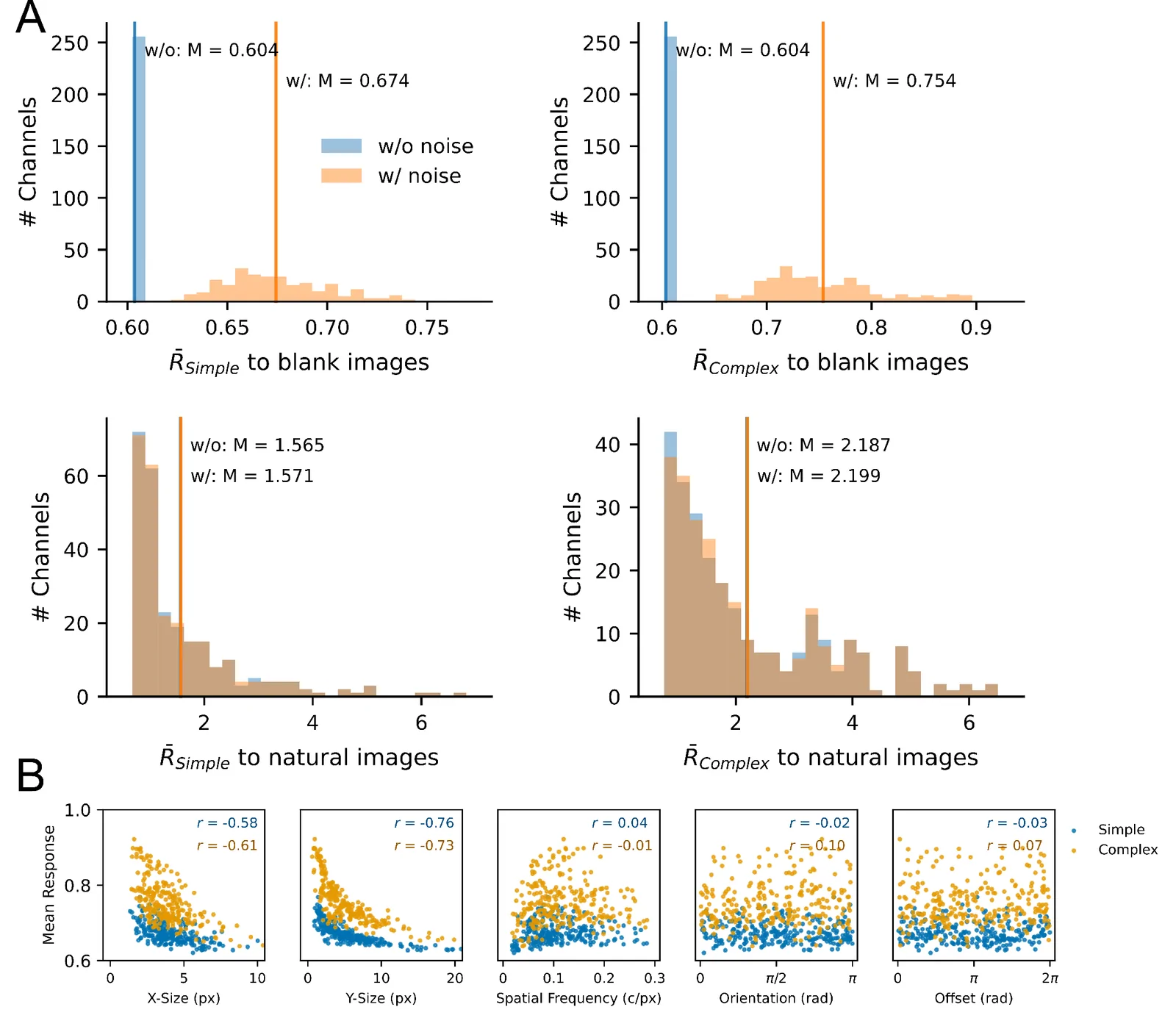
Distribution of simulated neural responses across different conditions and their relation to neuron properties. (**A**) Histograms of mean responses from individual channels. Compared to blank images without noise, those with noise tend to increase the variance of responses across channels and induce a higher response on average. For natural images, though the differences were small, pairwise comparison showed a clear difference (Supplementary Figure S1). (**B**) Relation between mean responses to noise images (y-axis) and the properties of the neuron (x-axes). Each dot represents a model neuron. Negative correlations were observed between sizes of channels and the mean responses of channels.

The observed channel responses are variable in part because of the external noise input, but also because of the internal noise in the simulated neurons. The internal noise followed a Gaussian distribution centered around zero with a variance equal to the mean response, approximating spike rates governed by a Poisson process (Dapello et al., 2020), but because of the nonlinear output functions, half-rectification, and squaring, only the positive portion of this distribution was reflected in the model output.

Images containing only external noise added to a blank image elicited higher neural responses compared to blank images (Figure 2A top; compare the distribution of simple and complex neural responses between “blank” and “blank + noise”). Responses to natural images also increased when visual noise was added, though this was primarily visible for channels with the lowest responses (Figure 2A, bottom). Because the signals from noise only images are clearer, for subsequent analyses, we focus on those.

As mentioned above, the size of receptive fields should in theory affect their sensitivity to external visual noise. To test this possibility, we plotted the mean response of neurons as a function of different RF properties (Figure 2B). The two indices of receptive field size were negatively correlated with neural responses (Figure 2B, first and second columns), aligning with the expectation that smaller receptive fields were more sensitive to the noise (X-size, *r* = −0.58, −0.61, for simple and complex channels respectively; Y-size, *r* = −0.76, −0.73, for simple and complex channels respectively, all *p* < 0.001). Spatial frequency was not correlated to the responses (*r* = 0.04, *p* = 0.55 for simple channels and *r* = −0.006, *p* = 0.928 for complex channels). And, as expected for isotropic noise, orientation and phase of the Gabor were independent of responses (n.s.).

Our next question was whether the spatially independent visual noise would result in independent neural responses in the simulated primary visual cortex. To test this hypothesis with maximal power, we again examined visual noise on blank backgrounds. We tested for correlations between different channels, pooling across space, i.e., receptive field locations: For a pair of channels, we correlated the pattern of responses across image locations. Figure 3A shows an example with x indexing the first channel response level (“channel A”) and y indexing the second channel response level (“channel B”), and each point in Figure 3B represents a different location in the image.

**Figure 3.**
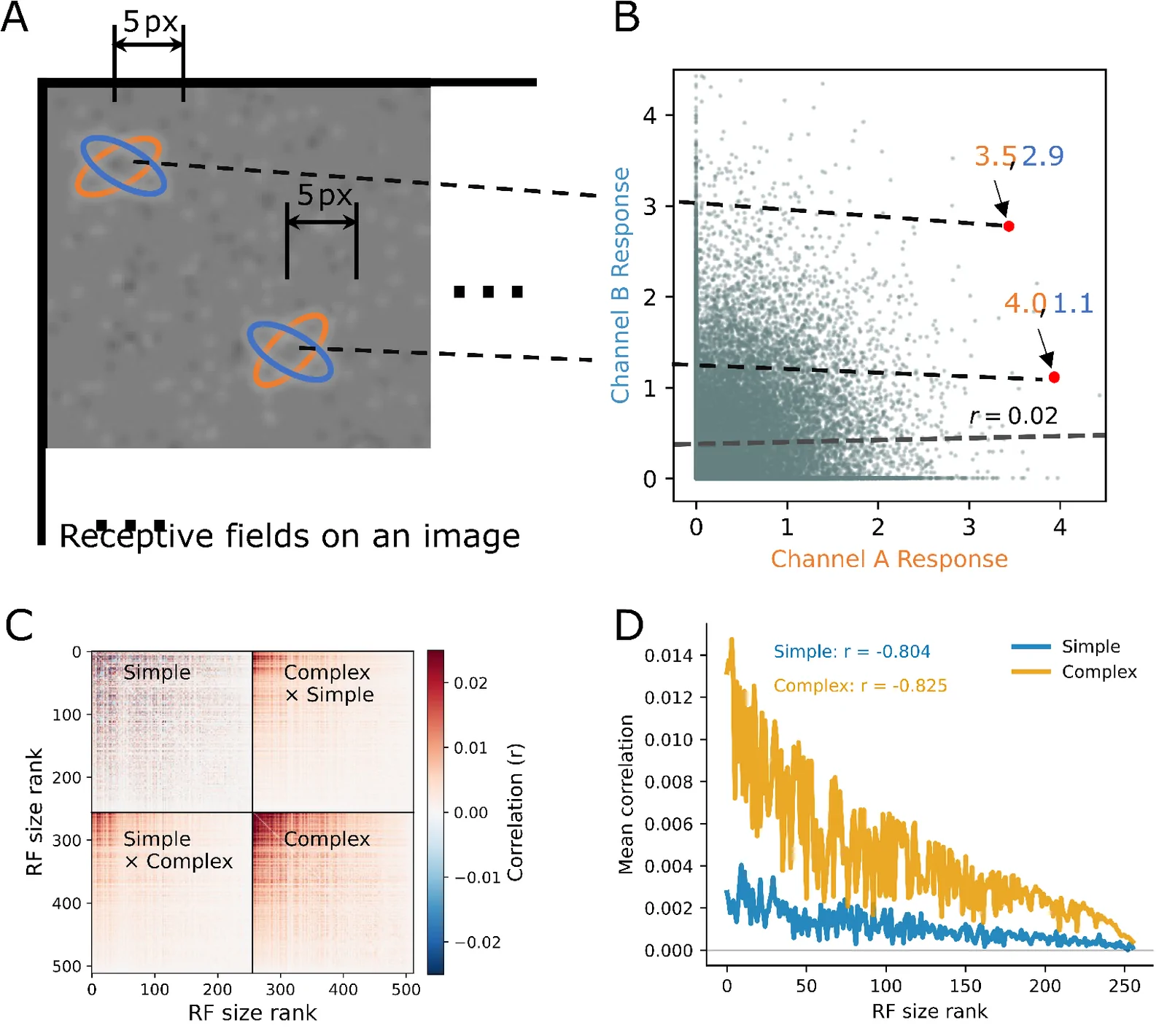
Correlations in neural responses. (**A**) Two example channels, outlined in orange and blue, at different locations on an image. (**B**) Scatterplot of the responses of the two overlapping channels in (**A**), with each dot representing a location on the image. Orange and blue numbers indicate the Channel A and B responses, respectively. The scatter plots were then used to calculate the correlation coefficient (Pearson *r* = 0.02 for this example) between channels. (**C**) Correlations in model neural responses were visualized based on the type of the channel, and the size of the neurons, when the neurons were at the same position. (**D**) Mean within-type correlation for each channel in (**C**), computed by averaging correlations with all other channels of the same type (simple or complex). Correlation coefficients between mean within-type correlation and RF size rank are indicated.

Pearson's correlations for each pair of channels are shown as pixels in the squares in Figure 3C, calculated as the average correlation across 100 repeats with random noise images. Correlations were small, but generally away from zero. Among simple channels, the mean absolute coefficient was $\bar{\left|r\right|}=0.0036$, and among complex channels, $\bar{\left|r\right|}=0.0044$. While pairs of simple channels had numbers of positive and negative correlations (27.6% significantly negative vs. 38.3% significantly positive, *t*-test across 100 images), pairs of complex channels were all positive (0.0% negative vs. 83.8% positive among correlations).

To confirm that the correlations were systematic and not just due to chance, we investigated effects of receptive field size on the correlations between channels. We defined sets of small and large sizes based upon a median split. For simple channels, mean absolute correlation between two small channels was substantially greater than that between two large channels ($\bar{|r_{small}|}=0.0066$ and $\bar{|r_{large}|}=0.0016$, For complex channels, an even larger difference was observed ($\bar{|r_{small}|}=0.0097$ and $\bar{|r_{large}|}=0.0017$). To further examine this relationship, we computed, for each channel, its mean pairwise correlation with all other channels of the same type (simple or complex), and plotted this quantity as a function of that first channel's receptive field size rank (Figure 3D). In both simple and complex channels, mean within-type correlation decreased systematically with channel rank, indicating that smaller receptive fields tended to exhibit stronger correlations than larger receptive fields (*r* = −0.804 and −0.825 for simple and complex channels, respectively). We also computed correlations between pairs of channels when they were separated spatially in the image (Supplementary Figures S2, S3). Channel spatial offsets reduced correlations, even for small offsets (e.g., 5 pixels), and all but eliminated them for larger offsets (≥10 pixels).

### Reconstruction of the images

To investigate the information encoded in the simulated neural populations, we decoded their signals by reconstructing the input images. The trained reconstruction network produced reasonably accurate reconstructions of both images with visual noise and without visual noise (Figure 4A).

We examined whether the spatial correlations between channels (Figure 3) were important for reconstructing the image. To answer this question, we tried to reconstruct each image from model responses that had been decorrelated (Figure 4B). For this analysis, we used only blank-background images containing external noise, because the effects of noise were clearer in this condition. We decorrelated the responses by shuffling channel responses across different images. Specifically, every activation in each of the 512 channels was replaced by the activation of the same channel drawn from a different randomly selected noise image. This shuffling procedure removed correlations between channels while preserving the marginal activation distribution within each channel. This decorrelation approach substantially reduced the visual noise in the reconstructed images (Figure 4B), indicating that inter-channel correlations are required for accurate reconstruction of noise inputs.

**Figure 4.**
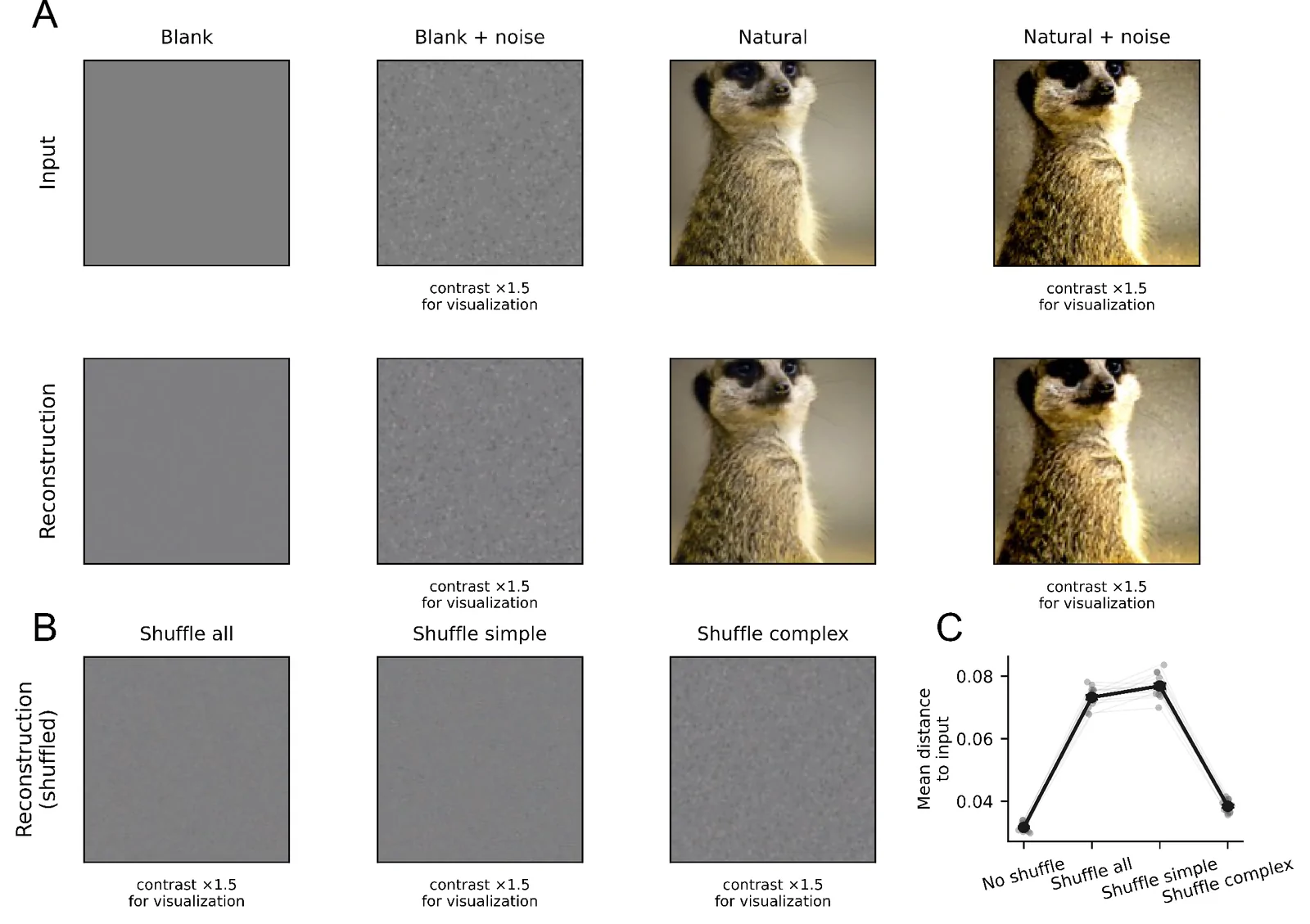
Example results of reconstruction. (**A**) Comparison between the external input (*top*) and the reconstructed images (bottom). Under all conditions, the reconstruction net could reconstruct visual noise on both natural images and blank images. (**B**) Comparison between reconstruction with all channels correlations intact and reconstruction with channels decorrelated by shuffling. (**C**) Difference in Portilla–Simoncelli texture statistics between reconstructions and the input noise across 100 samples. Manhattan distance (L1 norm) was used to summarize differences between the two sets of Portilla-Simoncelli statistics. Faint gray dots represent 16 example images selected from the full set of 100 images.

Simple and complex neurons exhibited different internal correlation patterns (Figure 3), with complex channels showing stronger correlations (especially with other complex channels). This suggests that correlations among complex neurons may play a greater role in encoding visual noise. To test this, we selectively decorrelated simple and complex channels, by shuffling their responses across images, and reconstructed the encoded images. Contrary to our expectation, only decorrelating simple cells noticeably impaired the reconstruction of external noise, whereas decorrelating complex cells had little effect (Figures 4B, 4C). To quantify the differences in reconstruction accuracy, we compared reconstructed images using Portilla–Simoncelli texture statistics (see Methods), which measures image features relevant for texture perception. Reconstructions obtained after shuffling simple channels showed the largest deviation from the original reconstructed noise pattern (*M* ± *SD* = 0.0769 ± 0.0034, *N* = 100 random visual noise images; Figure 4C), closely followed by shuffling all channels (*M* ± *SD* = 0.0732 ± 0.0029). Shuffling complex channels produced substantially smaller changes (*M* ± *SD* = 0.0384 ± 0.0019), and the unperturbed reconstruction showed the smallest deviation overall (*M* ± *SD* = 0.0317 ± 0.0013). This quantitative analysis confirmed that disrupting correlations among simple channels altered reconstructed visual-noise representations substantially more than disrupting correlations among complex channels.

To verify that this effect was not simply due to greater robustness of complex-cell representations to shuffling, we conducted an additional control analysis in which either simple or complex channels were selectively silenced without introducing decorrelation (Supplementary Figure S4). Consistent with the shuffling results, silencing complex channels impaired reconstruction less than silencing simple channels, indicating that complex channels contribute less to encoding the structure necessary for reconstructing visual noise patterns.

Reconstruction after shuffling all channels or shuffling simple channels did not produce completely blank images. Rather, these conditions produced very low-contrast noise patterns (Supplementary Figure S5). However, the resulting patterns were more colorful and less punctate than the noise in the original image, the noise in the reconstructed image (without shuffling), and the reconstructed noise when shuffling complex channels. These factors were reflected in the differences in Portilla–Simoncelli texture statistics discussed above.

## Discussion

We used a biologically-inspired neural network to simulate V1 neuronal responses to visual noise images. We found that noise induced distinctive patterns of responses in simulated V1 neurons, and, as expected, neurons with smaller receptive fields showed larger responses to noise. Importantly, spatially independent visual noise induced correlated responses between simulated neurons, and these correlations were critical for accurately reconstructing the noise images.

Our results contrast with retinal processing of visual noise, where correlations contribute minimally to information encoding (Meytlis et al., 2012; Pillow et al., 2008). This difference may be because receptive fields in the retina are generally substantially smaller than corresponding receptive fields in V1 (DeVries & Baylor, 1997; Rust et al., 2005). It may also stem from distinct patterns of neural organization. The tiled arrangement of retinal ganglion cells (DeVries & Baylor, 1997; Jun, Field, & Pearson, 2021) contrasts with the more extensively overlapping receptive fields of cortical neurons, and this overlap is likely to be important for producing the observed correlations in cortex. As receptive fields became less overlapping, as reflected by the distance between them, the observed correlations became weaker (Supplementary Figure S2, S3).

Naturalistic visual textures are characterized by relatively complex features, e.g., parallel oriented contours in a texture perceived as fur, and these features produce strong correlations between V1-like filters (Freeman et al., 2013). These correlation patterns may then be read out or transformed into more explicit perceptual representations in downstream visual areas such as V2, which is known to be sensitive to naturalistic texture statistics. Whether correlations induced by visual noise are represented similarly to naturalistic texture-related correlations remains unknown. Notably**,** phase-scrambled images—which are visually more similar to noise than natural textures—produce substantially weaker V2 responses (Freeman et al., 2013). At the same time, our results suggest that even visually random noise produces structured population correlations in V1, despite lacking obvious higher-order spatial organization. Hence, correlation-based coding of textures in V1 may extend beyond naturalistic textures. However, downstream representation of noise-like patterns may differ, potentially producing only weak enhancement in V2 engaging distinct representational mechanisms.

It is important to emphasize that in our model the observed correlations were mostly due to overlap between channel receptive field shapes (i.e. common feedforward input), as opposed to common input from feedback or lateral connections (Cohen & Kohn, 2011). Correlated activity arising from these other sources—such as those produced by attention, arousal, or global gain fluctuations—may also shape the representation of stimuli.

A related question is whether internally generated noise alone can give rise to structured noise-like percepts. To test this possibility, we manipulated the internal stochastic noise intensity in the V1 model while presenting a blank input image. When internal noise intensity was set to its physiologically plausible default level, the resulting activation distribution was similar to that produced by external visual noise input, but the perceptual noise was not visible unless the visualization contrast was boosted by a large factor (Supplementary Figure S6). Further increasing internal noise intensity also produced noise-like patterns in the reconstruction. However, both internally-generated sets of patterns were more colorful and had lower spatial frequency than the input noise. This is likely because independent internal noise alone does not produce the structured correlations required to generate noise patterns resembling our external visual noise inputs.

Our results also suggest a differential contribution of simple vs. complex cells to encoding external visual noise. Although both cell types showed correlations in their responses, decorrelating simple cell responses had a more detrimental effect on noise reconstruction. This differs from encoding of natural textures, which seems to depend most strongly on the responses of complex cells (Freeman et al., 2013; Ziemba, Freeman, Movshon, & Simoncelli, 2016). A possible explanation is that the objective function used during training the reconstruction algorithm biased the algorithm to focus on lower order features that are not translationally invariant. That is, the reconstruction net was optimized to reproduce the specific noise pattern, instead of its more invariant statistics, such as autocorrelation (Portilla & Simoncelli, 2000). For naturalistic textures, the information required for such a point-by-point reconstruction is lost by V2 (Ziemba & Simoncelli, 2021).

Our results have implications for understanding visual snow syndrome. The finding that correlations among model V1 neurons are crucial for encoding visual noise suggests that alterations in these correlation patterns could potentially contribute to perception of visual noise. For example, patients with visual snow may experience higher levels of correlated V1 neural activity (vs. typically sighted individuals) driven by *internal* noise from the retina or lateral geniculate nucleus, leading to an enhanced visibility of (typically imperceptible) noise. One way this could arise may be high gain in the input layers of V1, which could amplify noise from the retina to produce correlated responses in V1 among the neurons that share retinal inputs. If such correlations are insufficiently suppressed, they may contribute to the conscious perception of visual snow.

Potential evidence consistent with this possibility comes from reports of increased gamma-band activity in V1 in visual snow syndrome, although this finding may also reflect enhanced activity more generally rather than increased correlated activity specifically (Hepschke et al., 2022). Although visual snow is experienced dynamically, the spatial correlation structure may form the "single-frame" substrate that when extended over time by dynamic inputs, generates the percept of flickering visual noise. Current treatment approaches for visual snow symptoms show limited efficacy (van Dongen, Waaijer, Onderwater, Ferrari, & Terwindt, 2019; Eren & Schankin, 2020; Lukáčová, Mastík, & Minks, 2018; Puledda et al., 2022; Rusztyn, Stańska, Torbus, & Maciejewicz, 2023). If snow depends in part on correlated activity across V1 neurons, novel treatment approaches that seek to disrupt correlated activity (e.g., transcranial magnetic stimulation) may prove advantageous.

Alternatively, it remains possible that visual snow could arise from internal noise originating within V1 itself that fails to be suppressed from perception. Although amplified V1 internal noise reconstructions were less punctate and more colorful than our input noise, some snow patients may perceive particularly dense and colorful noise (Montoya et al., 2024).

Our findings may also inform psychophysical studies using noise stimuli. Many previous paradigms have used visual noise as a tool to eliminate or mask low level representations of preceding stimuli (e.g., Bacon-Macé et al., 2005), or coexisting stimuli (e.g., Pelli et al., 2003). Our results suggest that visual noise may induce certain patterns of V1 responses that could interact with the representation of stimuli.

One limitation of the present simulations is that the spatial scale of the visual noise elements was fixed relative to receptive field size. Because receptive field size and cortical magnification vary across eccentricity, different noise scales may engage different neural populations and produce different correlation structures. Consistent with this possibility, increasing visual snow element size shifted responses toward channels tuned to lower spatial frequencies and weakened the relationship between receptive field size and response strength (Supplementary Figure S7).

Although the present simulations primarily used the original VOneBlock architecture, we additionally tested a version incorporating a divisive normalization added to the output of the original model (cf., Burg et al., 2021). We found that the qualitative pattern of results remained unchanged, including the emergence of correlated responses to visual noise and their importance for reconstruction (Supplementary Figures S8, S9).

To train our reconstruction network we used the multi-scale structural similarity metric (Wang et al., 2003). Future work could test different metrics, and it may be useful to base one upon human judgments of noise patterns; we note that perception of noise itself (i.e., a noise pattern as signal) is a relatively unstudied topic.

A limitation of the present work is that both the stimuli and model responses were static, whereas perceptual visual noise, such as in visual snow syndrome, may be dynamic. Future work should incorporate temporal dynamics to test how time-varying correlations shape noise perception and the potential of various factors to produce visual snow. Another direction for future work would be to extend the model to incorporate additional factors known about other stages of visual processing. These include realistic retinal inputs to V1, including distinct magnocellular, parvocellular, and koniocellular streams, as well as later stages of processing including V2 (e.g., Ziemba & Simoncelli, 2021).

## Supplementary Material

Supplement 1
