# Supplementary material for: Perception of visual noise patterns depends on correlations produced in V1: A simulation study
Source: J Vis. 2026 Oct 9;26(10):10. doi: 10.1167/jov.26.10.10 (PMC13674664; doi:10.1167/jov.26.10.10)
Supplement: Supplementary file 1 [file jovi-26-10-10_s001.pdf]

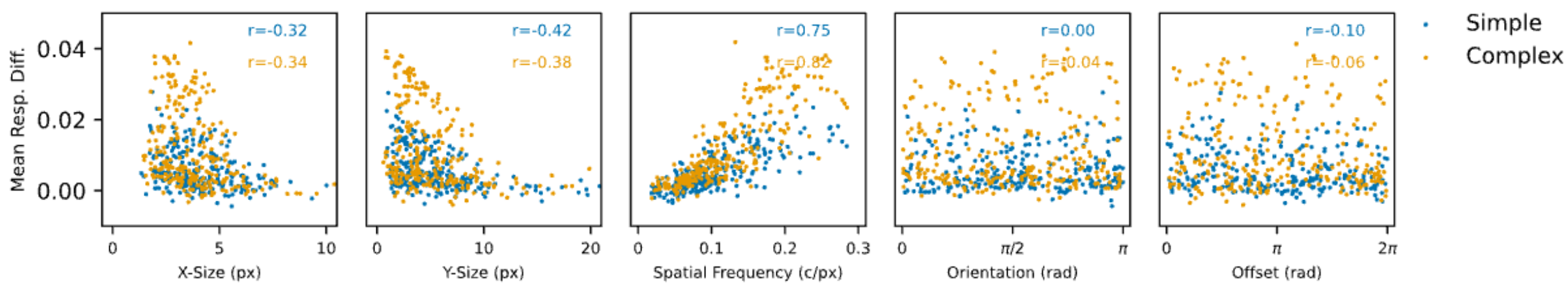

Figure S1. Effect of adding visual noise to natural images as a function of receptive field size. Average change in simulated channel response (noise-added minus original natural image) is plotted against Gabor receptive field parameters. Across channels, smaller receptive fields show larger response increases when visual noise is added, extending the main finding from blank backgrounds to naturalistic images.

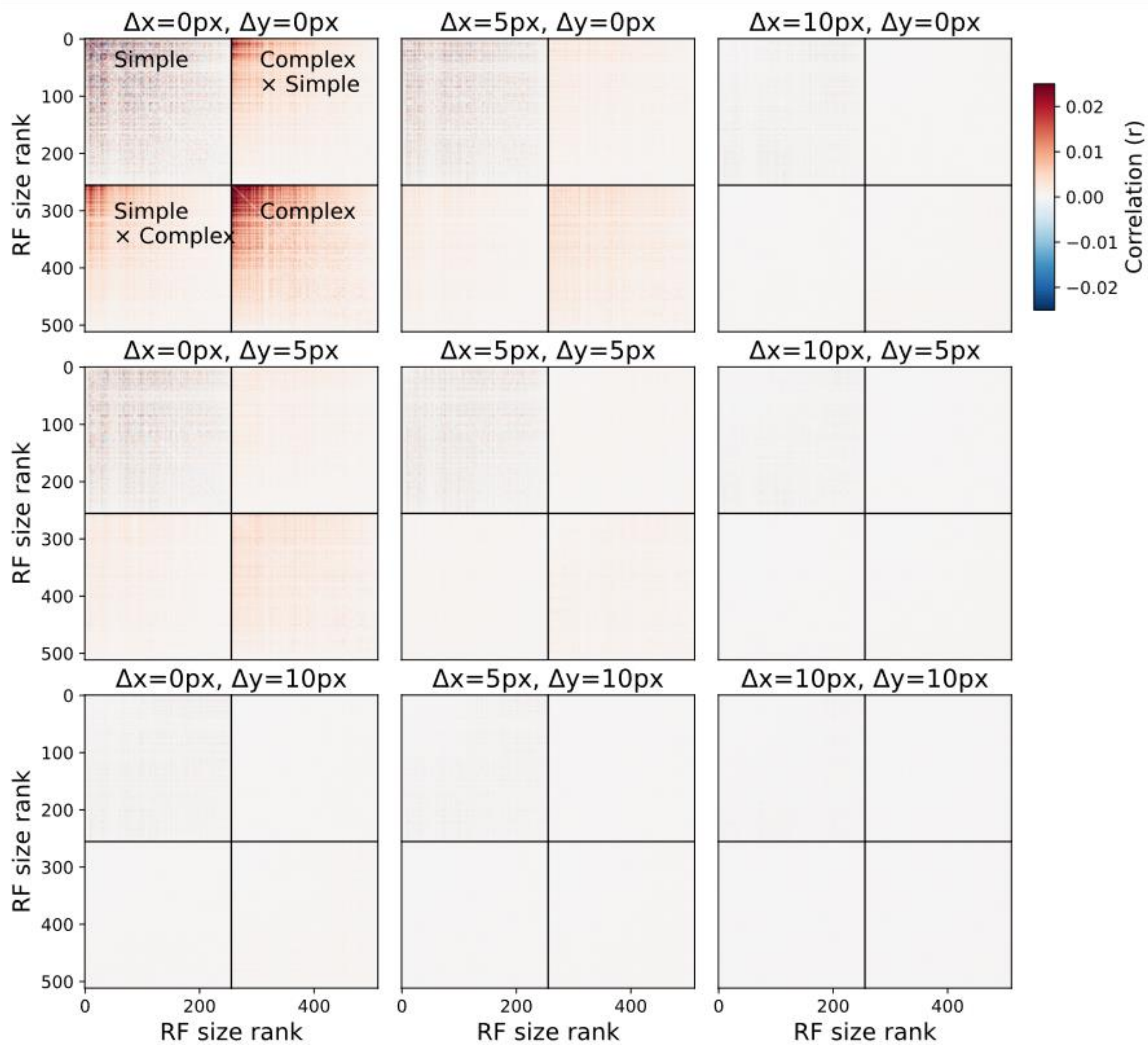

Figure S2. Correlation matrices for pairs of channels with spatial offsets. Each of the heatmaps represents a correlation matrix as in Figure 3C. Rows and columns of squares represent the vertical and horizontal difference in the position of two neurons between which correlation was calculated. For example, the top middle square represents a correlation matrix where the two cells in a pair were 5 pixels distant from each other horizontally. Correlations between simple and complex cells at the same position are reduced when one of the pairs is shifted spatially.

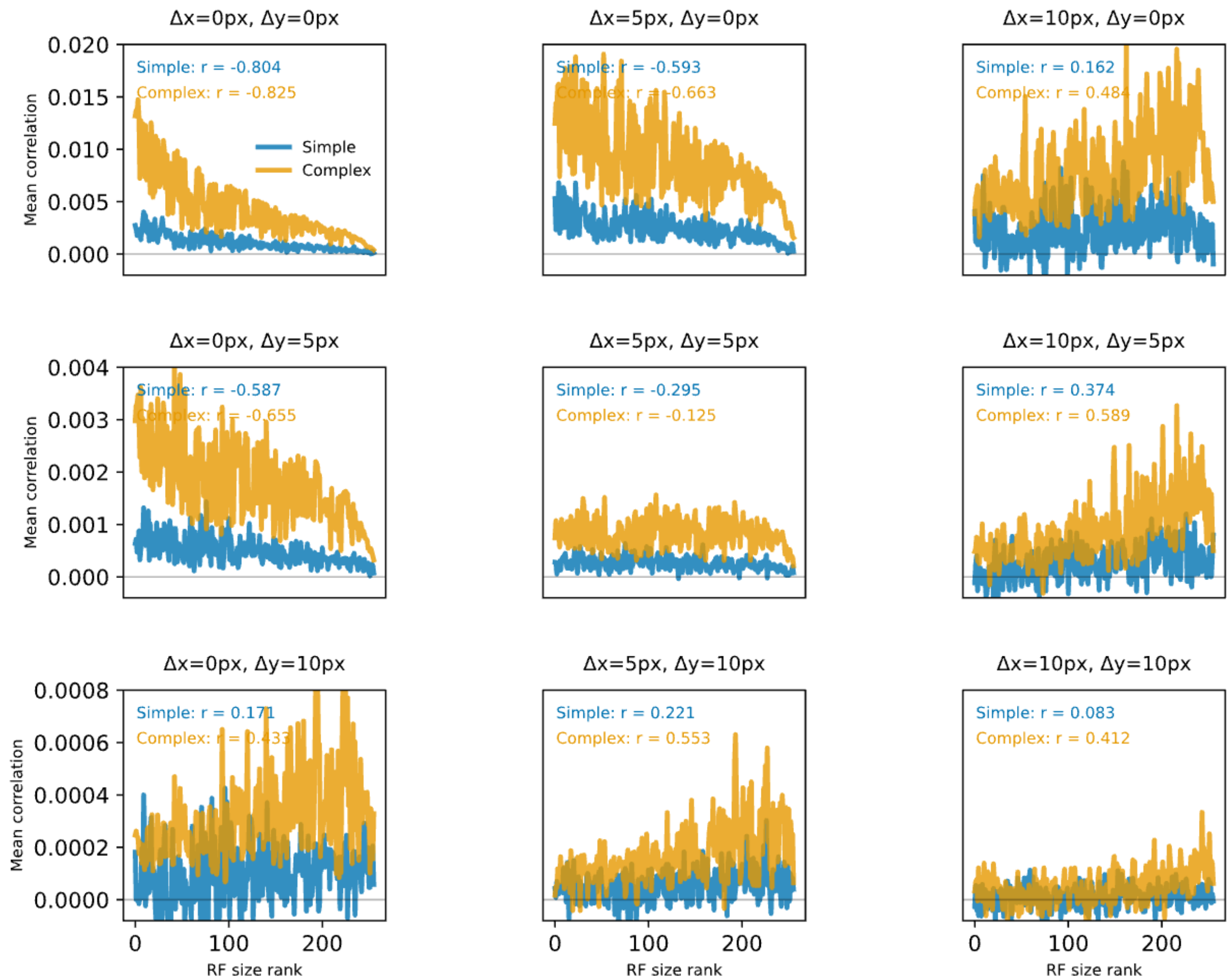

Figure S3. Averaged inter-channel correlations as a function of Gabor receptive field size, with spatial offsets. For each channel, average stimulus-driven correlation was computed across all channel pairs involving that channel and plotted against receptive field size rank. Panels additionally show how correlation structure changes when channel positions are spatially shifted relative to one another. Correlations are strongest for overlapping or nearby receptive fields and decline with increasing spatial offset.

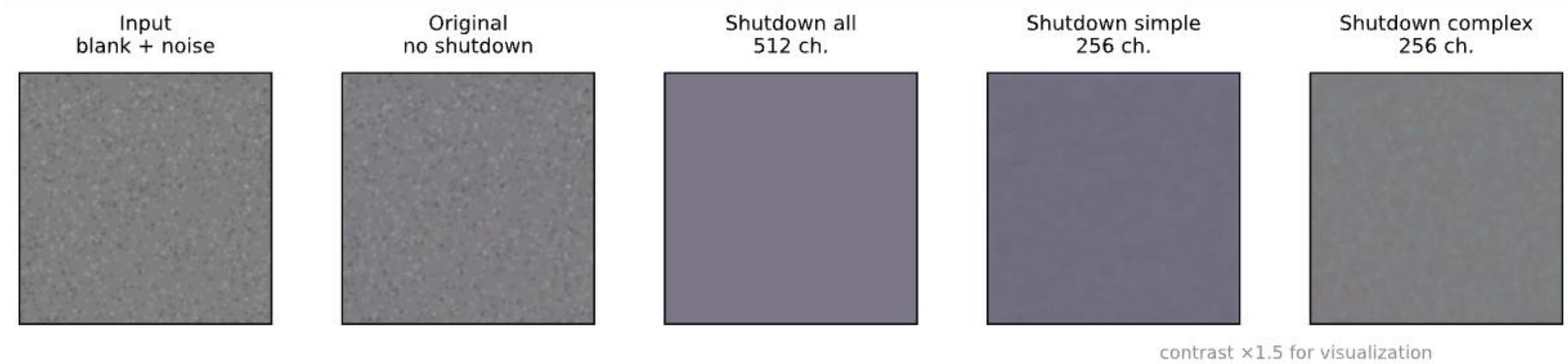

Figure S4. Reconstruction results under selective channel shutdown perturbations. Instead of shuffling responses, either simple cell or complex cell channels were selectively silenced by setting their activity to zero. Suppressing simple channels produced substantially larger degradation in reconstructed visual noise patterns than suppressing complex channels, supporting the conclusion that simple cell populations contribute more strongly to encoding fine spatial noise structure.

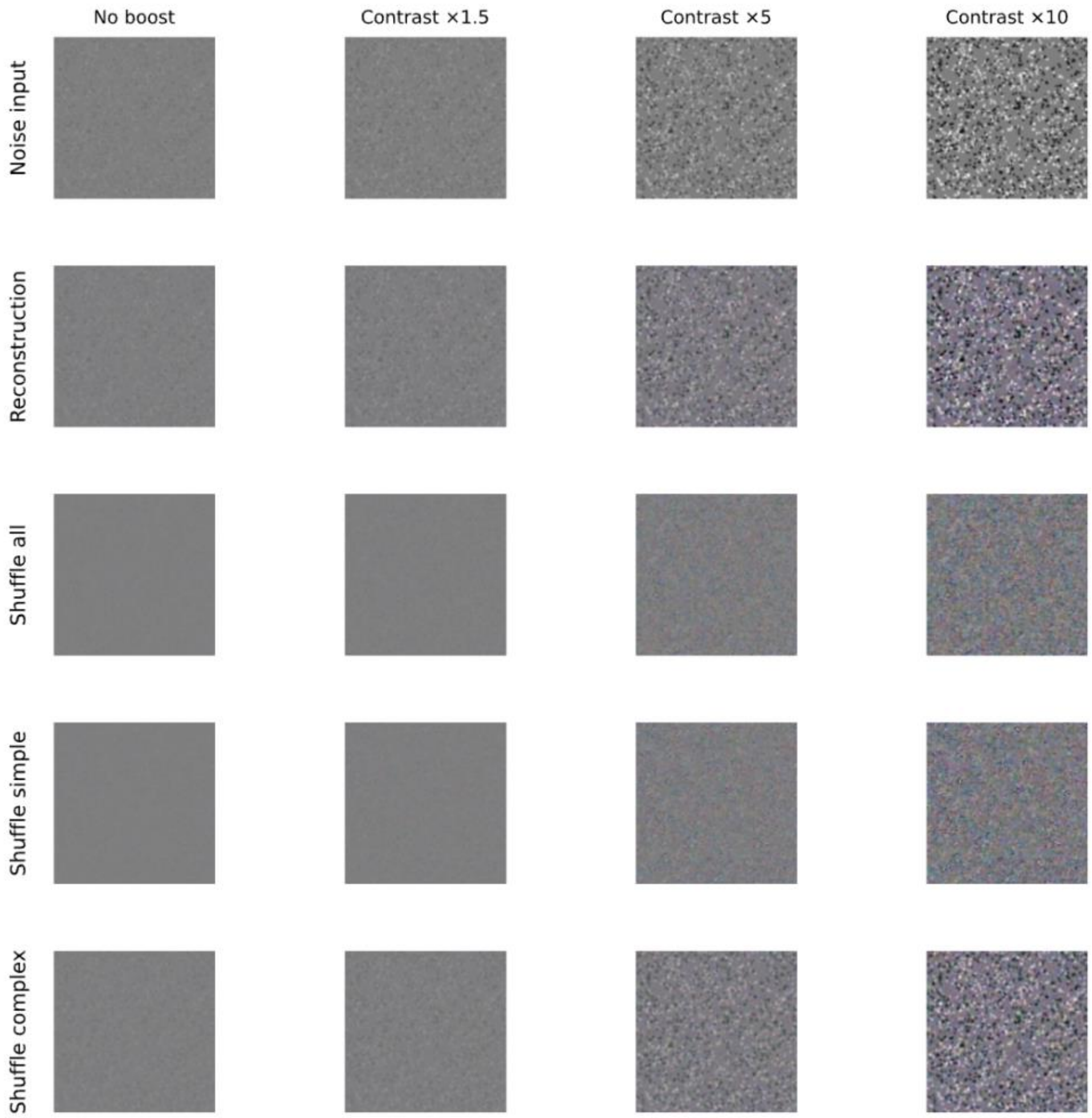

Figure S5. Visualization of reconstructed visual noise patterns (also shown in Figure 4) at different contrast boosting levels. Visual noise was presented on a blank input image, and reconstructed outputs from the V1 model are shown under increasing enhanced display contrast (×1, ×1.5, ×5, and ×10). At native contrast, reconstructed noise patterns were weakly visible, but contrast enhancement progressively revealed structured spatial patterns in the reconstruction. Shuffling across all channels, or across simple channels only, substantially disrupted the structured appearance of the reconstructed noise pattern. As contrast boost increased, reconstructions after shuffling all channels or simple channels also revealed visible noise patterns; however, these noise elements exhibited a colorful appearance unlike that of the input noise and were more densely distributed, both of which are reflected in the Portilla-Simoncelli statistics presented in main Figure 4.

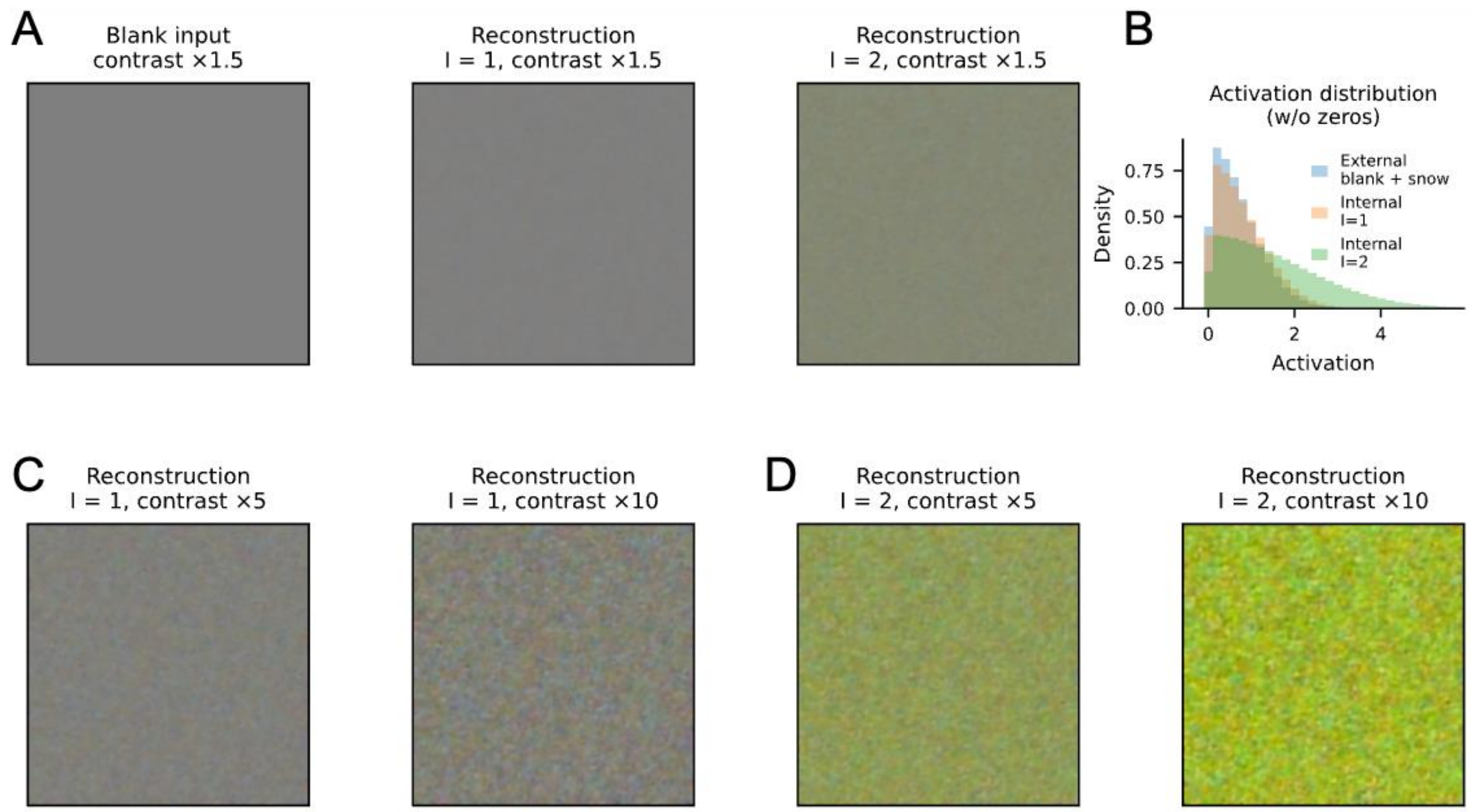

Figure S6. Effect of increasing internal stochastic noise in the V1 simulation. Internal noise was manipulated according to $y=x+\epsilon\max(sx+l,0)$, where $\epsilon \sim N(0,\sigma^2)$ denotes stochastic neuronal noise, s denotes noise scale, and l denotes noise level (adapted from Dapello et al., 2020). Here, *s* and *l* were fixed at their default values of ones, and only noise intensity (σ) was increased. When noise intensity was set to its default level of one, the resulting marginal activation distribution was similar to that produced by external visual noise input, but no perceptually visible noise pattern emerged in reconstructions from blank inputs unless image contrast was substantially boosted. Further increasing noise intensity produced noise-like reconstructed patterns, but these exhibited a systematic color bias and less punctate appearance than the perceptual appearance of visual snow. The color bias likely reflects an imbalance in the distribution of color-selective channels in the V1 model, rather than a property of internally generated noise itself. These results suggest that elevated local internal noise alone is insufficient to generate organized spontaneous activity resembling visual snow in the current feedforward model, and that additional mechanisms may be required to produce visual snow–like percepts.

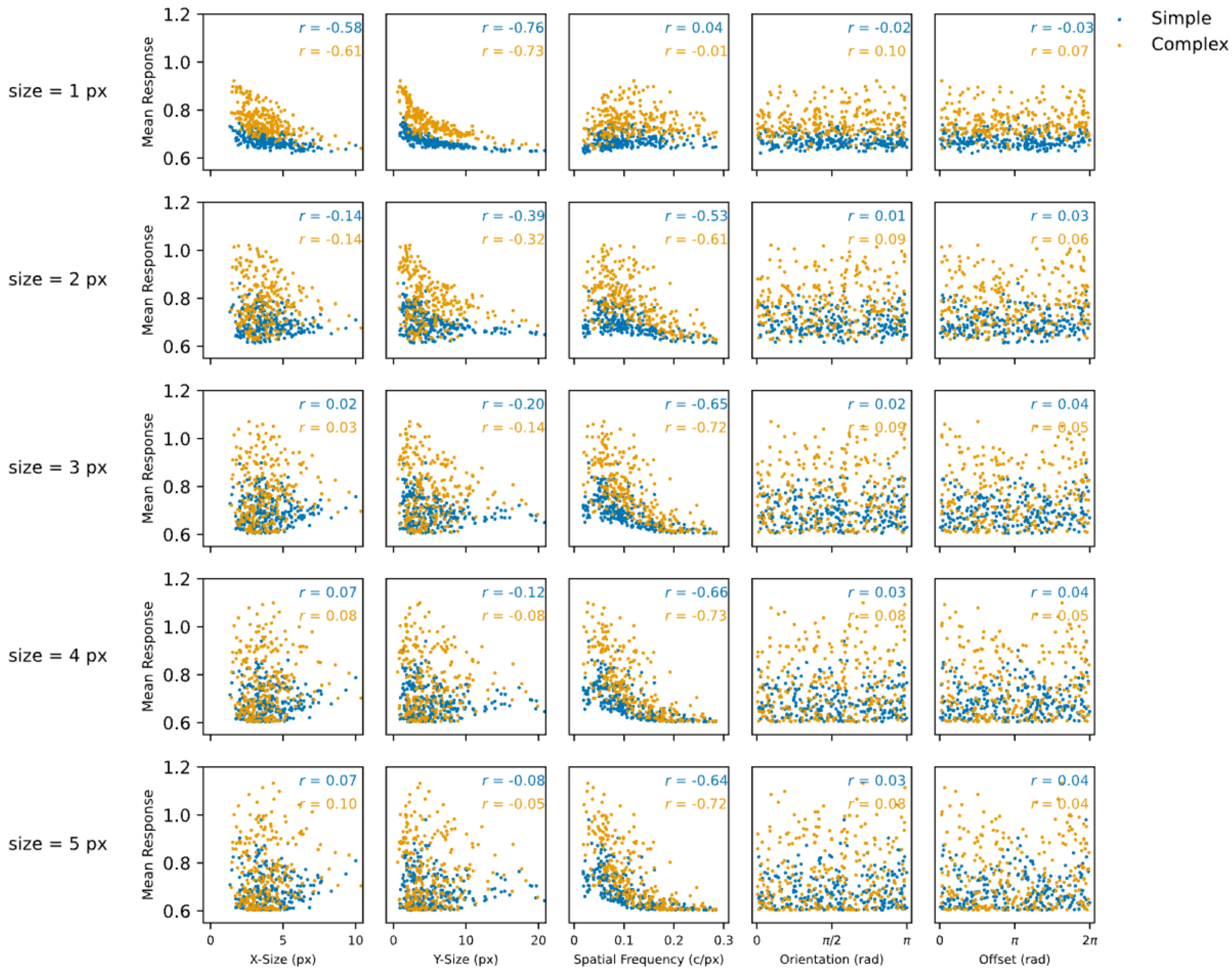

Figure S7. Dependence of simulated V1 neural responses on Gabor parameters across different visual snow element sizes. The relationship between mean channel response and receptive field properties is shown for noise elements ranging from 0.01 to 0.05 degrees of visual angle. As noise element size was increased, the negative relationship between response magnitude and receptive field size weakened, and the association with preferred spatial frequency reversed, consistent with larger noise elements preferentially driving channels tuned to coarser spatial structure.

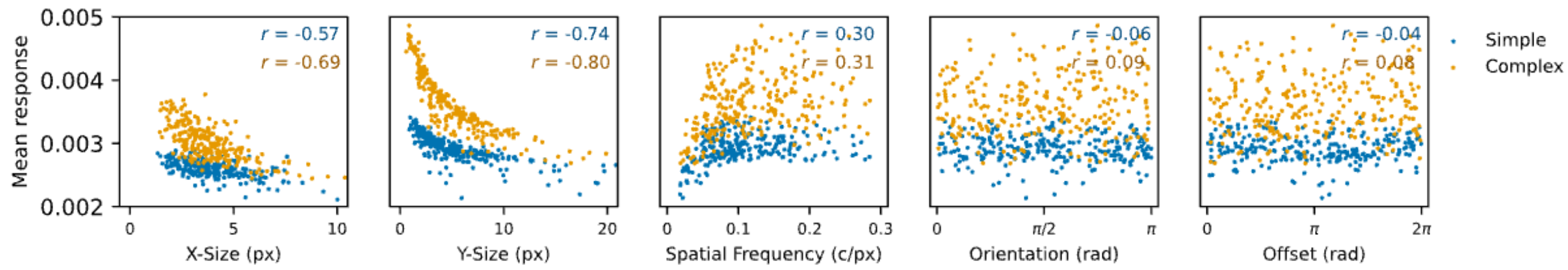

Figure S8. Response dependence on Gabor parameters in a model incorporating divisive normalization. Using a modified V1 model with divisive normalization, the same analysis as in Figure 2B shows that smaller receptive fields still exhibit stronger responses to visual noise. The qualitative pattern is preserved, demonstrating that the principal findings do not depend on omission of normalization.

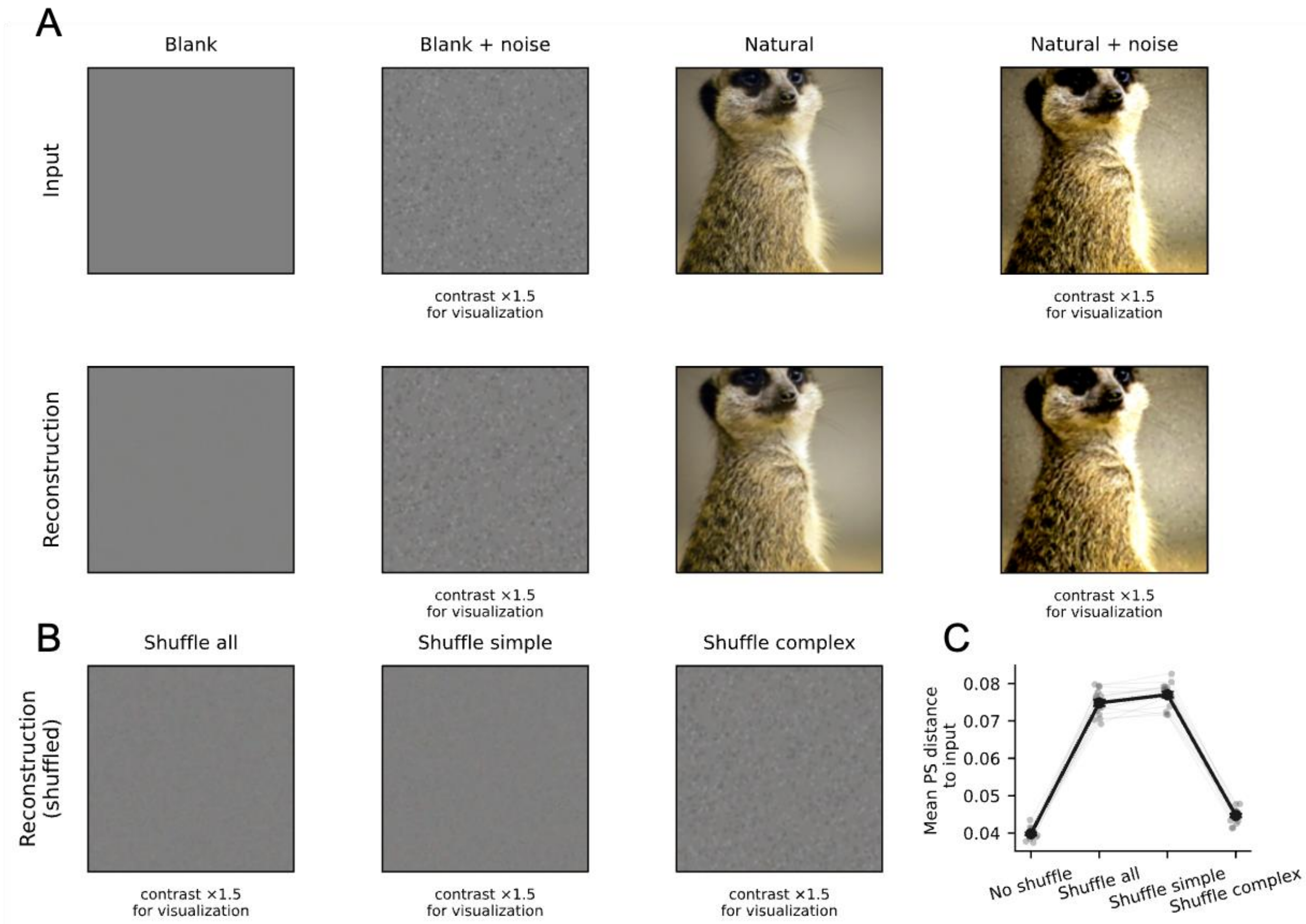

Figure S9. Reconstruction analyses using the divisive normalization model. For the same analysis framework as Figure 4, reconstructions remain sensitive to decorrelation manipulations, and disrupting inter-channel correlations degrades reconstruction of visual noise patterns. The persistence of this effect confirms that correlation-dependent encoding is robust to inclusion of divisive normalization.

References:

Burg, M. F., Cadena, S. A., Denfield, G. H., Walker, E. Y., Tolias, A. S., Bethge, M., & Ecker, A. S. (2021). Learning divisive normalization in primary visual cortex. *PLoS computational biology, 17*(6), e1009028.

Dapello, J., Marques, T., Schrimpf, M., Geiger, F., Cox, D., & DiCarlo, J. J. (2020). Simulating a primary visual cortex at the front of CNNs improves robustness to image perturbations. *Advances in Neural Information Processing Systems*, *33*, 13073-13087.
